# From Local to Global Modeling for Characterizing Calcium Dynamics and Their Effects on Electrical Activity and Exocytosis in Excitable Cells

**DOI:** 10.3390/ijms20236057

**Published:** 2019-11-30

**Authors:** Francesco Montefusco, Morten G. Pedersen

**Affiliations:** 1 Department of Information Engineering, University of Padova, 35131 Padova, Italy; pedersen@dei.unipd.it; 2 Department of Mathematics “Tullio Levi-Civita”, University of Padova, 35131 Padova, Italy; 3 Padova Neuroscience Center, University of Padova, 35131 Padova, Italy

**Keywords:** mathematical modeling, Ca^2+^ dynamics, ion channel complex, electrical activity, exocytosis

## Abstract

Electrical activity in neurons and other excitable cells is a result of complex interactions between the system of ion channels, involving both global coupling (e.g., via voltage or bulk cytosolic Ca^2+^ concentration) of the channels, and local coupling in ion channel complexes (e.g., via local Ca^2+^ concentration surrounding Ca^2+^ channels (CaVs), the so-called Ca^2+^ nanodomains). We recently devised a model of large-conductance BK_Ca_ potassium currents, and hence BK_Ca_–CaV complexes controlled locally by CaVs via Ca^2+^ nanodomains. We showed how different CaV types and BK_Ca_–CaV stoichiometries affect whole-cell electrical behavior. Ca^2+^ nanodomains are also important for triggering exocytosis of hormone-containing granules, and in this regard, we implemented a strategy to characterize the local interactions between granules and CaVs. In this study, we coupled electrical and exocytosis models respecting the local effects via Ca^2+^ nanodomains. By simulating scenarios with BK_Ca_–CaV complexes with different stoichiometries in pituitary cells, we achieved two main electrophysiological responses (continuous spiking or bursting) and investigated their effects on the downstream exocytosis process. By varying the number and distance of CaVs coupled with the granules, we found that bursting promotes exocytosis with faster rates than spiking. However, by normalizing to Ca^2+^ influx, we found that bursting is only slightly more efficient than spiking when CaVs are far away from granules, whereas no difference in efficiency between bursting and spiking is observed with close granule-CaV coupling.

## 1. Introduction

Mathematical modeling has played an important role in characterizing the electrical properties of neurons and other excitable cells. In this field, Hodgkin and Huxley were the first to propose a mathematical model for explaining ionic mechanisms underlying the generation and propagation of action potentials (APs) giant squid axons [1]. In the Hodgkin–Huxley model and most of its descendants, the system of ion channels is coupled globally via the membrane potential or the bulk cytosolic Ca^2+^ concentration. However, some ion channels are colocalized, implying that the activity of one channel may affect the other via local control: there is increasing evidence for direct coupling between certain ion channels and Ca^2+^ channels (CaVs) forming ion channel complexes [2,3,4]. A prominent example of ion channel complexes is the BK_Ca_–CaV complex: large-conductance, Ca^2+^-activated K^+^ channels (BK_Ca_ channels), ubiquitously expressed in excitable cells determining the electrical behavior [3], form ion channel complexes with CaVs, with a stoichiometry of 1–4 CaVs per BK_Ca_ channel [3,4]. Thus, the whole-cell population of BK_Ca_ channels is regulated both by global coupling, via the membrane potential, and by local coupling, via the Ca^2+^ nanodomains below the mouth of the CaV channels [5,6,7,8], where the local Ca^2+^ concentration reaches the tens of μM that are required for activating the BK_Ca_ channels at physiological voltages [3,9]. Cox [10] derived a Markov chain (MC) model for characterizing the single BK_Ca_–CaV complex with 1:1 stoichiometry, providing important insight into the open probability of BK_Ca_ channels during depolarizations and action potentials; for example, showing how inactivation of CaVs directly influence BK_Ca_ channel activity. Recently, by applying MC theory [11], reaction-diffusion models [10] and time scale analysis [12] to a more realistic BK_Ca_–CaV complex with 1:*n* stoichiometry, we [13] obtained a mechanistically correct model of the BK_Ca_ current, which respects the local effects of BK_Ca_–CaV coupling, and can be inserted in Hodgkin–Huxley-type models of whole-cell electrical activity: different CaV types and BK_Ca_–CaV stoichiometries affect BK_Ca_ channel activity and the resulting whole-cell electrical activity in neurons and other excitable cells. This kind of local-global modeling is similar to previous work on Ca^2+^ dynamics in cardiac cells [14].

Here, we present a study on how different electrophysiological behaviors determine the downstream Ca^2+^-regulated exocytosis process in endocrine cells, by which hormones are released from cells. In most endocrine cells, hormones are contained in granules that, in response to a series of cellular mechanisms culminating with an increase in the intracellular Ca^2+^ levels, fuse with the cell membrane allowing the release of their content (i.e., hormones) into the extracellular environment. The main mechanisms regulating hormone exocytosis are shared with exocytosis of synaptic vesicles underlying neurotransmitter release in neurons [15,16]. Different proteins mediate the process; in particular, the soluble N-ethylmaleimide sensitive factor attachment receptor proteins (SNAREs) [17]: SNAP and syntaxin, which are located in the cell membrane and VAMP, also called synaptobrevin, inserted into the vesicle/granule membrane. The v-SNAREs (v for vesicle) contained in the granule can form, with t-SNAREs (t for target) inserted in the cell membrane, the so-called SNARE complex [15], driving fusion of the two membranes, which—in the case of endocrine cells—allows the hormone molecules contained in the granule to exit the granule and enter the blood stream. SNARE complexes interact with other proteins, notably, Ca^2+^-sensing proteins, such as synaptotagmins, which trigger exocytosis upon Ca^2+^ binding. Therefore, the local Ca^2+^ concentration at the Ca^2+^ sensor of the exocytotic machinery is a key factor determining the probability (rate) of exocytosis of the secretory granule [18,19,20]. Experimental evidence of local coupling between single granules and CaVs showed that the exocytosis rate of a single granule increases significantly when it is close to CaVs [21]. Recently, we developed a method [22] for characterizing the local interactions between the single granule and the surrounding CaVs by exploiting a strategy that is similar to the methodology devised for describing the BK_Ca_ current [13]: using absorbing MC models allows for achieving analytic results for the expected exocytosis rate of a single granule, showing how coupling different numbers of CaVs at difference distances with the granule determines different responses.

In the following, we use the devised BK_Ca_ current model [13] as an example for analyzing the behavior (i.e., electrical activity) of a biologically realistic and important system (i.e., an endocrine pituitary cell) composed of units (i.e., ion channels) that interact both globally and locally: varying the number of CaVs per BK_Ca_–CaV complex results in different electrophysiological responses of pituitary cells, in particular, continuous spiking and so-called pseudo-plateau bursting, the latter characterized by few small oscillations riding on a depolarized plateau. Then, we couple electrical activity and exocytosis by combining the devised models [13,22] in order to investigate how local BK_Ca_–CaV coupling via continuous spiking or bursting in pituitary cells determine the downstream exocytotic response, by varying the distance and number of CaVs coupled with the single granules. In particular, we exploit MC models for describing the local coupling between CaVs and BK_Ca_-channels, and between CaVs and granules, and stationary approximations for characterizing the local Ca^2+^ levels, which allow us to couple electrical activity and exocytosis in a straightforward manner. Moreover, we reduce model complexity to achieve simplified ordinary differential equation (ODE) models that respect the local control by CaVs of BK_Ca_ channels and granules.

## 2. Results

### 2.1. Whole-Cell Electrical Activity Modeling Respecting Local Control

Local coupling of ion channels is important in determining whole-cell activity. BK_Ca_–CaV complexes provide an example of ion channel complexes, where the BK_Ca_ activity is influenced locally by associated CaVs, and differences in stoichiometry of the complex (1–4 CaV channels per BK_Ca_ channel [3,4,23]) can affect not only single channel activity but also whole-cell behavior significantly [13].

A model of pituitary cells has been used to study the role of BK_Ca_ channels in the generation of so-called plateau bursting, which consists of a few small oscillations riding on a depolarized plateau, and is important for secretion [24,25]. In the original model [26] the BK_Ca_ current was modeled as a purely voltage-dependent current, neglecting Ca^2+^ dependency. In [13], we substituted this simplified representation of the BK_Ca_ current with a concise model that respects the local effects of BK_Ca_–CaV coupling and can be inserted in the Hodgkin–Huxley-type model of electrical activity of pituitary cells (see Methods, Equation (6)). For characterizing the whole-cell BK_Ca_ current $I_{BK}$, the state of every single BK_Ca_ channel does not need to be known; it is sufficient to determine the BK_Ca_ open probability $p_{Y}^{\left(n\right)}$ over time, where the superscript *n* indicates the number of CaVs coupled with the single BK_Ca_ channel, and $I_{BK}$ in Equation (6) has the the following form:

(1) $$I_{BK}=g_{BK}p_{Y}^{\left(n\right)}\left(V-V_{K}\right)\;.$$

#### 2.1.1. BK_Ca_–CaV Complex with 1:1 Stoichiometry

**Figure 1:**
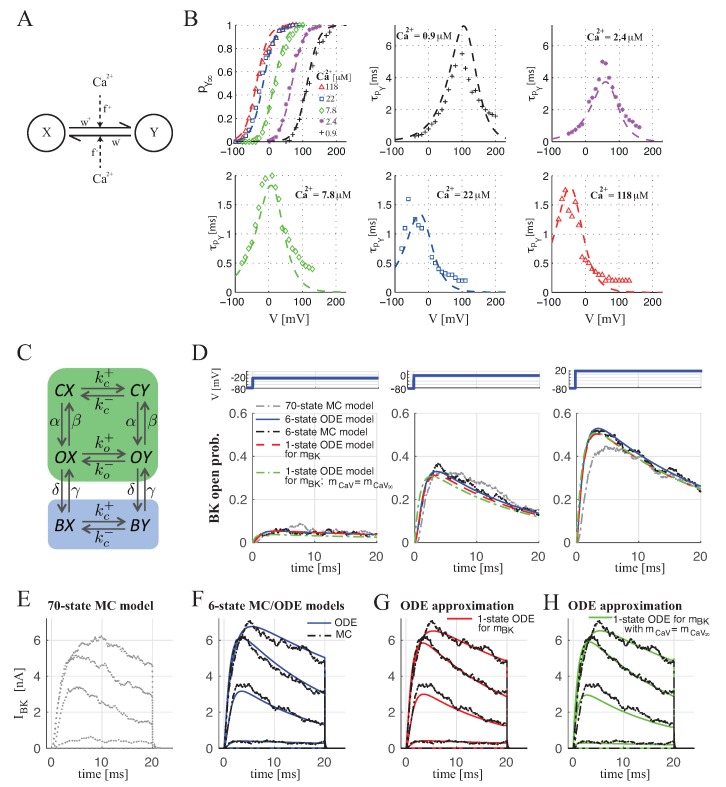
Modeling of BK_Ca_–CaV complex with 1:1 stoichiometry. (**A**) Schematic representation of the BK_Ca_ model, where *X* and *Y* indicate the closed and open states of the BK_Ca_ channel. (**B**) Fit to the experimental data [10] of the 2-state model of panel A: steady-state BK_Ca_ open probabilities versus voltage at different Ca^2+^ concentrations (different markers for different Ca^2+^ levels as indicated in the legend) and the corresponding fit obtained by the model (dashed lines) (upper left plot); time constants (in ms) versus voltage data at given Ca^2+^ concentrations (see legend in each plot), and the corresponding model fits (dashed lines). (**C**) Scheme indicating the six states of the devised MC model for BK_Ca_–CaV complex with 1:1 stoichiometry. CX, OX and BX correspond to the closed state for the BK_Ca_ channel (*X*) coupled with the closed (*C*), open (*O*) and inactivated (*B*) states for the CaV, respectively, and CY, OY and BY correspond to the open state for the BK_Ca_ channel (*Y*) coupled with the closed (*C*), open (*O*) and inactivated (*B*) states for the CaV, respectively. The green box indicates states with non-inactivated CaV, whereas the blue box highlights states with inactivated CaV. (**D**) Simulated open probabilities, in response to three different voltage steps, from −80 mV to −20 (left), 0 (middle) and 20 mV (right), for BK_Ca_ channels controlled by CaVs in complexes with 1:1 stoichiometry, obtained from different models: the original 70-state Markov chain model (gray; [10]); the 6-state Markov chain model (shown in panel C—black); the ODE model corresponding to the 6-state model (blue; Equation (12)); the simplified Hodgkin–Huxley-type model, $p_{Y}^{\left(1\right)}=m_{BK}^{\left(1\right)}h$, where $m_{BK}^{\left(1\right)}$ is given by Equations (13)–(15) (dashed red) and the corresponding model assuming instantaneous activation $m_{CaV}=m_{CaV,\infty }$ with $m_{BK}^{\left(1\right)}$ defined by Equation (13) with the approximations of Equations (16) and (17) (dash-dotted green). (**E**–**H**) Simulated BK_Ca_ currents in response to the different voltage steps (from −80 mV to from −40 mV to 40 mV in 20 mV increments for 20 ms and then back to −80 mV) obtained from the different models as in **D**. In **D**–**H** the average of one-thousand Monte Carlo simulations for each MC models is shown.

In the case of BK_Ca_–CaV complex with 1:1 stoichiometry, in order to compute $p_{Y}^{\left(1\right)}$, we devised a 6-state MC model obtained by coupling a 2-state model for the BK_Ca_ channel (Figure 1A), whose parameters are set by fitting the available experimental data [10] (see Methods and Figure 1B) and a 3-state model for the CaV channel (Figure S1C). By assuming the corresponding 6-state ODE model and using time-scale analysis (in particular, CaV inactivation is slow compared to other processes), we split the model in two submodels, one including states with non-inactivated CaV (green box in Figure 1C) and the other with inactivated CaV (blue box in Figure 1C). Moreover, the submodel describing the BK_Ca_ channel activation in BK_Ca_–CaV complex with non-inactivated CaV, denoted with $m_{BK}^{\left(1\right)}$, can be described by one ODE of Equation (13) (see Methods)—a quasi-steady state approximation. The BK_Ca_ channels in BK_Ca_–CaV complexes exhibit inactivation because of inactivation of the associated CaVs, and with approximately identical dynamics, as found in experiments [27] and Monte Carlo simulations (see Figure 1; [10]). Then, the open probability for BK_Ca_–CaV complex with 1:1 stoichiometry, $p_{Y}^{\left(1\right)}$, can be expressed as

(2) $$p_{Y}^{\left(1\right)}=m_{BK}^{\left(1\right)}h\;,$$

with $m_{BK}^{\left(1\right)}$ given by Equation (13) and *h* represents the inactivation function of the CaVs (see Methods). We make a further simplification assuming instantaneous CaV activation, since in many whole-cell models (e.g., [26,28,29,30]), the Ca^2+^ currents are assumed to activate instantaneously: in this case, i.e., instantaneous CaV activation, $m_{CaV}=m_{CaV,\infty }$, where $m_{CaV}$ and $m_{CaV,\infty }$ represent CaV activation variable and its steady-state, respectively (see Equations (8) and (9)); $m_{BK}^{\left(1\right)}$ is given by Equation (13) with the approximations defined by Equations (16) and (17); and h=1−b with *b* by Equation (10) (see Methods).

As performed in [13], we compared the devised 6-state MC model of the BK–CaV complex with the first stochastic model of the gating of this complex devised by Cox [10], obtained by coupling the 10-state MC model for the BK_Ca_ channel with a 7-state MC model for the CaV channel (see Methods and Figure S1), resulting in a 70-state MC model. The simulated open probabilities were very similar for both the models in response to different voltage steps, from −80 mV to −20, 0 and 20 mV (compare the dotted gray curve (70-state MC model) with the dash-dotted black one (6-state MC model)). Moreover, the open probability obtained by the 6-state ODE model (the solid blue curve) approximates well, the average fraction of open channels calculated from Monte Carlo simulations of the corresponding 6-state MC model, and the open-probability expression $m_{BK}^{\left(1\right)}h$ of Equation (2) (dashed red curve). Additionally, the further simplification assuming instantaneous CaV activation (dash-dotted green curve; $m_{BK}^{\left(1\right)}$ given by Equation (13) with the approximations of Equations (16) and (17)) approximates the full system decently, except for the initial phase before CaV activation reaches equilibrium. Finally, Figure 1E–H shows how the simplified BK_Ca_ models reproduce, satisfactorily, the whole-cell BK_Ca_ currents $I_{BK}$ for each step in voltage ($I_{BK}$ defined by Equation (1) with n=1, where $g_{BK}=N_{BK}{\bar{g}}_{BK}$, with ${\bar{g}}_{BK}=100$ pF [31] the single-channel conductance and $N_{BK}=1000$ the number of BK_Ca_ channels): the simplified 6-state Markov chain model (black plots in Figure 1F–H) and the corresponding 6-state ODE model (blue plots in Figure 1F) approximate the 70-state Markov chain model current very well (Figure 1E; [10]); the simplified Hodgkin–Huxley-type model current for the BK_Ca_ channel with $p_{Y}^{\left(1\right)}=m_{BK}^{\left(1\right)}h$ (Equation (2); red plots in Figure 1G), and the corresponding model assuming instantaneous activation of the CaV currents ($m_{BK}^{\left(1\right)}$ given by Equation (13) with the approximations of Equations (16) and (17); green plots in Figure 1H) also work very well.

#### 2.1.2. BK_Ca_–CaV Complex with 1:*n* Stoichiometry

**Figure 2:**
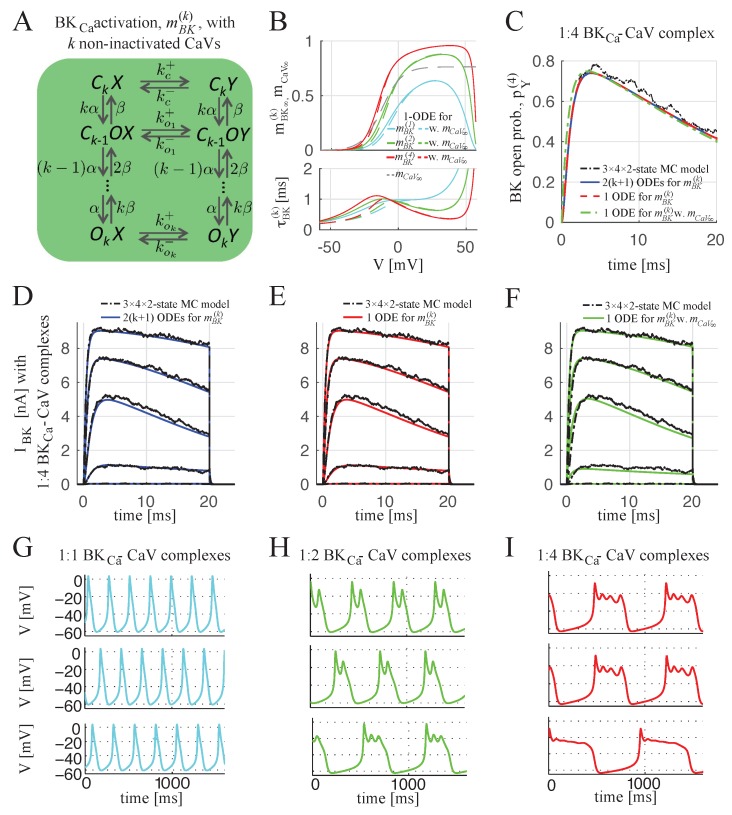
Multiple CaVs per BK_Ca_–CaV complex: from local to whole-cell behavior. (**A**) Markov chain (MC) model for complexes with *k* non-inactivated CaVs. (**B**) Steady-state BK_Ca_ activation functions, $m_{BK}^{\left(k\right)}$ (upper plot), and time-constants, $\tau_{BK}^{\left(k\right)}$ (lower), for BK_Ca_ channels in complexes with k=1 (cyan), 2 (green) or 4 (red) CaVs, given from 1-ODE by (26) (solid) and from the simplification assuming $m_{CaV}=m_{CaV,\infty }$ by Equations (28) and (29) (dashed). The gray dashed curve shows the CaV activation function $m_{CaV,\infty }$, for comparison. (**C**) Simulated BK_Ca_ open probabilities, $p_{Y}^{\left(4\right)}$, for the case of 1:4 BK_Ca_–CaV stoichiometry, in response to a voltage step from –80 mV to 0 mV, obtained from the MC model of 3×4×2 states (black), from the $2\left(k+1\right)$ ODEs describing the states in panel **A** coupled to CaV inactivation ($p_{Y}^{\left(4\right)}$ defined by Equation (3) with $m_{BK}^{\left(k\right)}$ given by Equation (18)—solid blue), from the reduced ODE model considering CaV activation kinetics ($p_{Y}^{\left(4\right)}$ by Equation (3) with $m_{BK}^{\left(k\right)}$ given from 1-ODE by (26)— dashed red), and from the simplification assuming $m_{CaV}=m_{CaV,\infty }$ (Equation (26) for $m_{BK}^{\left(k\right)}$ with Equations (28) and (29)—dash-dotted green). (**D**–**F**) Simulated whole-cell BK_Ca_ currents, $I_{BK}$, defined by Equation (1) in response to the different voltage steps (as in Figure 1E–H) obtained from the different ODE models used for characterizing $m_{BK}^{\left(k\right)}$, as in (**C**): $m_{BK}^{\left(k\right)}$ given by Equation (18) (blue curves in panel **D**); $m_{BK}^{\left(k\right)}$ given by Equation (26) (red curves in panel **E**); $m_{BK}^{\left(k\right)}$ given by Equation (26) with assumptions of Equations (28) and (29) (green curves in panel **F**). Panels **C**–**F** show the average of one-thousand Monte Carlo simulations for the MC model of 3×4×2 states (black). (**G**–**I**) Simulated electrical activity in a model of lactotrophs [26] with 1:*n* BK_Ca_–CaV complexes, and with n=1 (**G**), n=2 (**H**) or n=4 (**I**). $I_{BK}$ is described by Equation (4), where $m_{BK}^{\left(n\right)}$ is modeled by Equation (18) (upper plots), Equation (26) (middle) and Equation (26) with assumptions of Equations (28) and (29) (lower).

In the case of more than one CaV per BK_Ca_ channel, the linear buffer approximation is used to compute the Ca^2+^ profile from *n* channels by superimposing *n* nanodomains found for single, isolated CaVs. Then, the MC model of Figure 1C can be extended to a model with 3×n×2 states. However, as discussed previously, we assumed that, on a fast timescale, the fraction *h* of non-inactivated CaVs is constant, and note that the BK_Ca_ channel closes rapidly when all CaVs in the complex are inactivated. Hence, for the case of 1:*n* BK_Ca_–CaV stoichiometry, we split the system according to the number *k* of non-inactivated CaVs: the BK_Ca_ activation can be described on a fast time scale by the Markov chain model of Figure 2A with 2×(k+1) states. As for the case of 1:1 stoichiometry, the dynamics of the BK_Ca_ open probability in complexes with *k* non-inactivated CaVs can be approximated by a single ODE (see $m_{BK}^{\left(k\right)}$ defined by (26)). Then, the open probability of the BK_Ca_ channel coupled with *n* CaVs, $p_{Y}^{\left(n\right)}$, can be estimated by taking into account that the probability of *k* non-inactivated CaVs being present in a complex with *n* CaVs, nkhk(1−h)n−k. Then, $p_{Y}^{\left(n\right)}$ can be expressed as

(3) pY(n)=∑k=1nnkhk(1−h)n−kmBK(k).

As performed in [13], we compared the simulated open probabilities obtained from the different models in response to a voltage step from −80 to 0 mV, and the results reported in Figure 2C show how the different ODE models exploited for computing $m_{BK}^{\left(k\right)}$ in Equation (3) (blue curve, $m_{BK}^{\left(k\right)}$ by Equation (18); red curve, $m_{BK}^{\left(k\right)}$ by Equation (26); green curve, $m_{BK}^{\left(k\right)}$ by Equation (26) with instantaneous CaV activation defined by Equations (28) and (29)) effectively approximate the Monte Carlo simulations of the full Markov Chain model with 3×n×2 states (black curve).

The whole-cell BK_Ca_ current defined by Equation (1), with $p_{Y}^{\left(n\right)}$ given by (3), involves the reduced model *n* ODEs (Equation 26) for the activation variables $m_{BK}^{\left(k\right)}$ with k=1,…,n. Note that if the *n* CaVs do not inactivate, Equation (1) reduces to

(4) $$I_{BK}=g_{BK}m_{BK}^{\left(n\right)}\left(V-V_{K}\right)\;,$$

which involves one ODE (Equation 26) for $m_{BK}^{\left(n\right)}$.

Figure 2D–F shows the simulated whole-cell BK_Ca_ currents $I_{BK}$ for the case of 1:4 BK_Ca_–CaV stoichiometry (defined by Equation (1), where $g_{BK}=N_{BK}{\bar{g}}_{BK}$, with ${\bar{g}}_{BK}=100$ pF [31] as in the previous section, while $N_{BK}$ is lower and equal to 700 in order to reproduce the experimental data; i.e., the maximum amplitude values reported in [10,32]) obtained from the different ODE models (exploited for computing $m_{BK}^{\left(k\right)}$) in response to different voltage steps and compared with the simulated BK_Ca_ currents obtained from 3×4×2 MC model (n=4). In all the cases, the different ODE models for characterizing $m_{BK}^{\left(k\right)}$ (Equation (18) (blue curves), Equation (26) (red curves) and Equation (26) with Equations (28) and (29) (green curves)) approximate very well the Monte Carlo simulations (black curve) obtained from the 3×4×2 MC model.

#### 2.1.3. Concise Whole-Cell Modeling Respecting Local Control

The Hodgkin–Huxley-type model of the BK_Ca_ current defined by Equation (1), that allows us to take into account the local interactions in BK_Ca_–CaV complexes, can be exploited to investigate how the stoichiometry of the complex affects whole-cell electrical behavior of pituitary cells.

In the case of BK_Ca_–CaV complexes with 1:1 stoichiometry, spiking electrical activity is observed as shown in Figure 2G, since insufficient BK_Ca_ current is generated. The different plots in Figure 2G (upper, middle and lower) correspond to different ODE models used for computing the open probability of the single complex $p_{Y}^{\left(1\right)}$ defined in Equation (1) for n=1: The 4-state ODE model (BX and BY are not considered since CaV does not inactivate) with $p_{Y}^{\left(1\right)}$ defined by Equation (12) and with $p_{BY}=0$ (upper). The single ODE model for $m_{BK}^{\left(1\right)}$ defined by Equation (13) with CaV kinetics leading to $p_{Y}^{\left(1\right)}=m_{BK}^{\left(1\right)}$ (Equation (2) since h=1) (middle), with simplifications for $m_{BK}^{\left(1\right)}$ of Equation (13) assuming instantaneous CaV activation by Equations (16) and (17) (lower).

In contrast, in the case of BK_Ca_–CaV complexes with 1:*n* stoichiometry with n>1, plateau bursting appears with the number of small oscillations per burst depending on the number of CaVs per BK_Ca_–CaV complex, as shown in Figure 2H,I for n=2 and n=4, respectively. The different plots in Figure 2H,I (upper, middle and lower) correspond to the different ODE models used for computing $m_{BK}^{\left(n\right)}$, and hence, the resulting $I_{BK}$ of Equation (4)—$m_{BK}^{\left(n\right)}$, given by Equation (18) (upper blue plots) by solving the complete ODE model of $2\left(k+1\right)$ equations; $m_{BK}^{\left(n\right)}$, given by single ODE of Equation (26) (middle curves); $m_{BK}^{\left(n\right)}$, given by Equation (26) with Equations (28) and (29) by assuming instantaneous CaV activation, $m_{CaV}=m_{CaV,\infty }$ (lower curves).

Although the quantitative behavior is independent of the approximation for $m_{BK}^{\left(n\right)}$, minor qualitative differences are present. The approximation given by Equation (26) reproduces very well, the behavior obtained from the complete ODE model for the activation of the BK_Ca_ channel surrounded by *n* non-inactivated CaVs defined by Equation (18) (Figure 2G–I, upper and middle panels), whereas the further simplification given by Equations (28) and (29) produces smaller and more spikes per burst (lower panels). Nonetheless, considering parameter uncertainties and experimental variations, even Equations (28) and (29) produce reliable results.

### 2.2. Coupling Electrical Activity with Exocytosis

**Figure 3:**
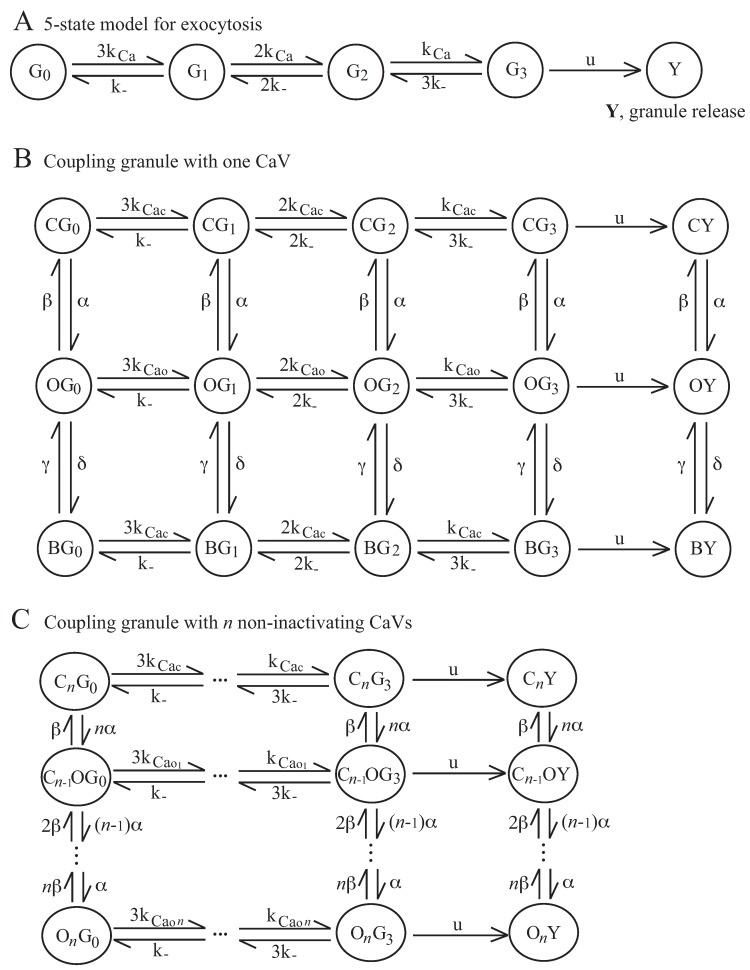
Models for exocytosis of single granule and granule-CaV complex. (**A**) The 5-state model for exocytosis of a single granule adjacent to the plasma membrane, where $G_{0}$ corresponds to the state with no bound Ca^2+^ ions, $G_{1}$ to one, $G_{2}$ to two and $G_{3}$ to three. (**B**) Model of the granule-CaV complex where the granule is described by the model shown in panel **A** and the CaV by the model shown in Figure S1A. (**C**) Model of the granule coupled with *n* non-inactivating CaVs, where the granule is described by the model shown in panel **A** and the CaV by a single-channel gating with two states, closed, *C*, and open, *O*.

We investigated how cellular electrical activity regulates the downstream exocytosis process. We modeled the single granule containing the hormones released from the cell by Ca^2+^-regulated exocytosis process with the 5-state MC model of Figure 3A: the granule can be in one of four states depending on the number of Ca^2+^ ions bound to the Ca^2+^ sensor on the granule (states $G_{i}$, with i=0,…3, representing the number of Ca^2+^ ions bound to the granule sensor) before fusing with the membrane and releasing its hormone content (state *Y*) (see Methods for more details). The Ca^2+^ concentration at the granule sensor, $Ca_{CaV}\left(r_{G}\right)$ given by Equation (7) with $r=r_{G}$ being the distance from the CaV pore to the Ca^2+^ sensor on the granule, drives the exocytosis MC model, allowing the granule to modify its state through the Markov chain from $G_{0}$ to *Y* and undergo exocytosis.

In the following, in order to characterize the local interactions between granules and CaVs, we analyzed the property of the secretory complex obtained by coupling a single granule with one or more CaVs: we coupled the granule, described by the 5-state MC model of Figure 3A, with *n* CaVs, each one described by the 3-state MC model of Figure S1A, and obtained the $\left(5\times n\times 3\right)$-state MC model, as developed in [22]. For the electrical activity model in pituitary cells defined by Equation (6), CaVs do not inactivate, and the exocytosis can be described by the $5\left(n+1\right)$-state MC model shown in Figure 3C. Using this model, the granule state depends on the CaVs states. In particular, for the case with one CaV, the Ca^2+^ concentration at the granule sensor needed for triggering exocytosis, is equal to a basal level ($Ca_{c}$) when the CaV is closed or inactivated, and is equal to $Ca_{CaV}\left(r_{G}\right)$ defined by Equation (7) when the CaVs is open; for a more general case with *n* CaVs, the linear buffer approximation [7] is used to summarize Ca^2+^ levels at the granule sensor when more than one CaV is open, as performed for BK_Ca_–CaV complex. In [22], we used phase-type distribution results for Markov chains [11] for estimating the expected exocytosis rate (the release probability) of a single granule. We found that the distance $r_{G}$ is a major factor in determining the exocytosis rate, as recently demonstrated and quantified explicitly [21]. Furthermore, and in agreement with the experiments [21], the results showed that increasing the number of CaVs coupled with the granule determines a much higher rise of the exocytosis rate, which in the case of inactivating CaVs is more pronounced when the granule is close to CaVs (about 10 nm), whereas for non-inactivating CaVs the highest relative increase in rate is obtained when the granule is far from CaVs (about 50 nm), suggesting that it is not necessary that the granule is very close to CaVs for triggering exocytosis. However, in [22] we did not take into account the coupling between electrical activity and exocytosis, assuming constant values for the membrane potential. Here, the Ca^2+^ dynamics, and hence, the CaVs states, are driven by the electrophysiological behavior, which depends on the local interactions between CaVs and BK_Ca_ channels, as shown in the previous section. In particular, we studied how the two typical electrophysiological behaviors (spiking or bursting), observed in pituitary cells due to different local coupling between CaVs and BK_Ca_ channels, determine the granule release by varying the number and the distance of CaVs coupled with the granules.

#### Spiking versus Bursting on Evoking Exocytosis

**Figure 4:**
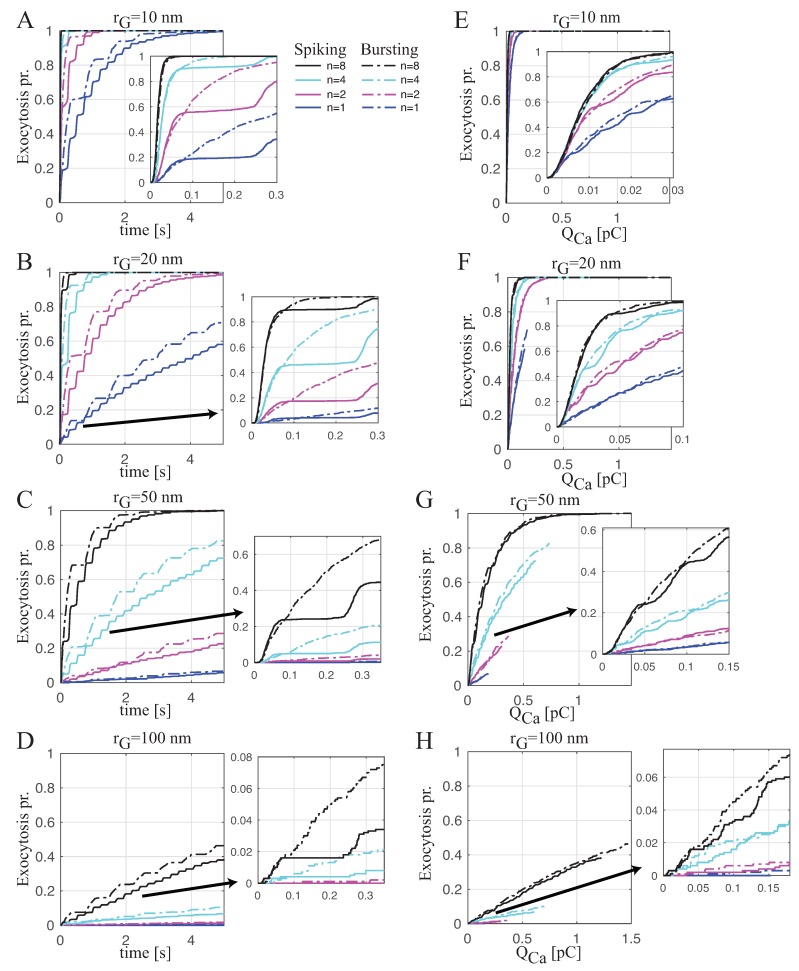
Probability of exocytosis for single granule coupled with *n* CaVs driven by spiking or bursting. (**A**–**D**). Probability of exocytosis versus time for the granule at fixed distances $r_{G}$ from *n* CaVs (n=1 (blue curves), n=2 (magenta curves), n=4 (cyan curves) and n=8 (black curves), driven by spiking (solid lines) or bursting (dash-dotted lines)): $r_{G}=10$ nm (**A**); $r_{G}=20$ nm (**B**); $r_{G}\;=\;50$ nm (**C**); $r_{G}=100$ nm (**D**). (**E**–**H**) Probability of exocytosis versus total Ca^2+^ influx, $Q_{Ca}$ (computed by integrating Ca^2+^ current over the time), for panels (**A**–**D**) respectively. Each panel shows the average of one-thousand Monte Carlo simulations, which were performed for the MC exocytosis model.

Figure 4A–D shows the time evolution of the exocytosis probability of a single granule coupled with different numbers of CaVs placed at difference distances. In each panel, for the granule coupled with *n* CaVs placed at fixed distance $r_{G}$, we assumed that CaVs dynamics are driven by continuous spiking in the form of Figure 2G (upper plot) or bursting, as in Figure 2H (upper plot). When the CaVs are close to the granule (see Figure 4A,B for $r_{G}=10$ and 20 nm, respectively), the bursting pattern (dash-dotted lines) evokes release at a higher rate than the spiking pattern (solid lines) for a reduced number of CaVs coupled to the granule (the blue and magenta lines for n=1 and 2, respectively), while this difference in the rate decreases when the number of CaVs increases (see the cyan and black lines for n=4 and 8, respectively). When the CaV are far from the granule (see Figure 4C,D for $r_{G}=50$ and 100 nm, respectively), bursting promotes exocytosis with faster rates than spiking even for a higher number of CaVs coupled to the granule. This difference in secretion rate between spiking and bursting is mainly due to larger amount of Ca^2+^ entering during bursting, which becomes negligible when the number of CaVs is high and close to the granule. In this scenario, the Ca^2+^ concentrations at the exocytotic machinery are in saturation regimes for both the electrical patterns.

**Figure 5:**
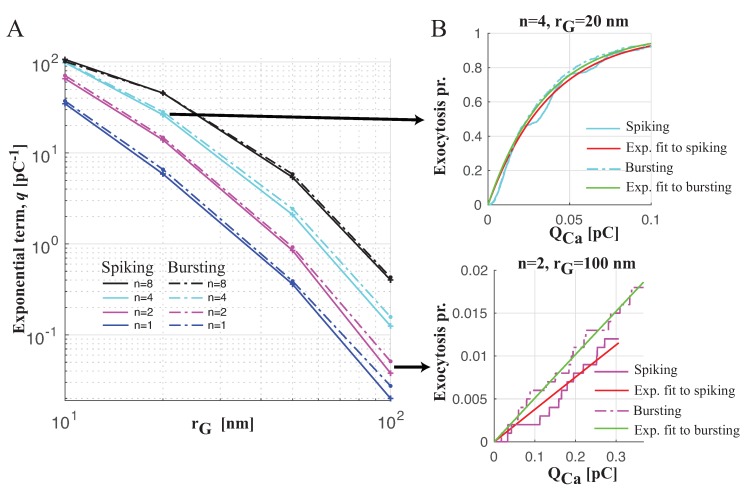
Fitting to the simulated exocytosis probabilities. (**A**) Trend (in logarithmic scale) of *q* parameter of function $f_{e}$ defined by Equation (5) with respect to the distance $r_{G}$ of the granule from *n* CaVs (n=1 (blue curves), n=2 (magenta curves), n=4 (cyan curves) and n=8 (black curves)), driven by spiking (solid lines) or bursting (dash-dotted lines). Note that for each estimate the 95% confidence interval is very limited. (**B**) Fit to the to the simulated exocytosis probabilities versus $Q_{Ca}$ for the granule at $r_{G}=20$ from four CaVs (upper plot) and for the granule at $r_{G}=100$ from two CaVs. The simulated responses are the averages of one-thousand Monte Carlo simulations.

Therefore, in order to evaluate the exocytotic machinery performance with the same Ca^2+^ entry, we analyzed the exocytosis probability with respect to the total Ca^2+^ influx, $Q_{Ca}$. Figure 4E–H shows the granule release probability versus $Q_{Ca}$ for the same cases simulated in Figure 4A–D. From this analysis, it is seems that the efficiency in evoking exocytosis between spiking and bursting is similar. This finding is also confirmed by fitting the simulated responses using an exponential function $f_{e}$ defined as

(5) $$f_{e}=1-e^{-q\;Q_{Ca}}\;,$$

with the aim to estimate the parameter *q*, whose value can provide insight into the efficiency in evoking granule release (a high value of *q* means high efficiency). Figure 5A shows the trend of *q* with respect to the distance $r_{G}$ of the granule from different numbers of coupled CaVs (different colors) for the two electrical patterns, spiking (solid lines) and bursting (dash-dotted lines). When the granule is close to CaVs, independently of their number, bursting and spiking have a similar effect on exocytosis, whereas, when the granule is far from CaVs, in the case of few CaVs (one or two), bursting is slightly more efficient than spiking. For all the cases, the fitting approximates the simulated data well, as shown for two cases in Figure 5B: the upper plot, reporting the simulated exocytosis probabilities of a granule at distance $r_{G}=20$ nm from four CaVs for spiking and bursting patterns, and the corresponding fitting shows how there is no virtually difference between the two electrical patterns in evoking exocytosis; the lower plot, displaying the simulated exocytosis probabilities of a granule at distance $r_{G}=100$ nm from two CaVs for spiking and bursting patterns and the corresponding fitting, shows how there is a small difference between spiking and bursting, with the latter resulting more efficient in granule release.

## 3. Discussion

In this paper, we show the role of mathematical modeling as an important tool for investigating excitable cells with focus on ion Ca^2+^ channels and their local interactions with BK_Ca_ potassium channels in influencing electrical activity of pituitary cells and with hormone-containing granules determining the granule release by exocytosis process. Therefore, whole-cell models have to be consistent with the local mechanisms operating at molecular levels, and hence, we show how to exploit all the available information in a coherent and structural way by using mathematical modeling in order to handle the complexity of cellular electrophysiology.

In order to handle the local interactions in BK_Ca_–CaV complexes with 1:*n* stoichiometry, we used a stochastic model based on Markov chain theory (of 3×n×2 states; see Figure 2), as a starting point for analyzing the single complex dynamics. However, the fluctuations resulting from stochastic gating kinetics observed at the molecular level tend to become negligible as a system’s size approaches the whole-cell scale, where, for describing the electrical behavior, it is not necessary to know the state of each single complex, but it is sufficient to know the open probability of BK_Ca_ channel population. Therefore, we used the corresponding deterministic model, and by exploiting time-scale analysis and quasi-steady state approximation, we achieved a concise, deterministic Hodgkin–Huxley-type model of BK_Ca_ currents defined by Equations (1) and (3). This approach allowed us not only to reduce model complexity and computational costs of the stochastic model, but also to achieve an explicit interpretation of the parameters and their effects on whole-cell behavior through the direct read of the formula of the simplified deterministic model: We showed that increasing the number of CaVs coupled with BK_Ca_ channel determines a left shift of the BK_Ca_ activation curve, since the probability of at least one CaV being open is greater with more channels in the complex (see Equations (28) and (29) assuming instantaneous activation of CaVs). We also derived in [13] an analytic expression for the time to first opening of a BK_Ca_ channel, providing theoretical insight into stochastic simulation results. Then, the concise Hodgkin–Huxley-type model of whole-cell BK_Ca_ currents, that allows taking into account local control by CaVs in BK_Ca_–CaV complexes, can be inserted into models of cellular electrical activity, showing, in particular, how different BK_Ca_–CaV stoichiometries cause different electrophysiological responses, including continuous spiking and bursting in pituitary cells. In [13], we also showed how BK_Ca_–CaV stoichiometry controls the fast after-hyperpolarization (fAHP) in a model of hypothalamic neurosecretory cells; i.e., the undershoot seen after an action potential controlling firing frequency and transmitter release [3,33]. Moreover, coupling BK_Ca_ channels with different CaV types affects electrical activity differently in human pancreatic beta-cells, and further insight into the control by CaVs of BK_Ca_ channels, whose block stimulates insulin secretion in human [34] and mouse [35] beta-cells, may lead to a better understanding of beta-cell function and how it becomes disturbed in diabetes. Furthermore, this approach should be relatively straightforward to apply to other ion channel complexes; e.g., the CaV3–Kv4 complex [2].

Recently, we [22] exploited the methodology devised for modeling the BK_Ca_ currents for handling the local interactions between granules and CaVs, and specifically, by using phase-type distribution results for Markov chains [11], we obtained analytic results for the expected exocytosis rate of a granule coupled with different numbers of CaVs placed at different distances. We also exploited a quasi-steady state approximation for the corresponding ODE model of the 5-state MC model for exocytosis of a single granule adjacent to the plasma membrane, as shown in Figure 3A, in order to reduce the model complexity, especially in the case of the granule coupled with *n* CaVs. However, in this case, the quasi-stead approximation only works for the final state of the chain (i.e., state $G_{3}$), before the granule can fuse with the cell membrane (i.e., state *Y*), since its dynamics are the fastest. The sequence of the different states of the MC for the granule before fusing, according to the number of Ca^2+^ ions bound to the Ca^2+^ sensor on the granule, allows us to reproduce the delayed exocytosis with respect to a raise in the calcium concentration, as observed by flash-release experiments [36,37,38], and in this study, we used the complete MC models of Figure 3 for describing granule exocytosis. Hence, by modeling the local Ca^2+^ dynamics, we coupled exocytosis and electrical models with the aim to investigate how the two main electrophysiological behaviors in pituitary cells, continuous spiking and bursting, affect the downstream exocytosis process. From our results, we found that, surprisingly, bursting is only slightly more efficient than spiking, and only when CaVs are few and far from the granule. These differences to previous findings [25] can be explained by the fact that bursting has an important role in the resupply of the primed granule pool, which depends on the global, rather than local, Ca^2+^ concentration. Indeed, as experimentally observed [39], global Ca^2+^ concentration (i.e., the bulk cytosolic Ca^2+^ concentration) is higher during bursting than spiking. We did not model the resupply, since we assumed that the granule was already primed for exocytosis.

For our aims, we used steady-state reaction-diffusion equations for characterizing Ca^2+^ levels, in particular, for calculating calcium concentrations at ion channel BK_Ca_–CaV complexes and at granules, as performed in previous works: Cox [10] found that, for computing Ca^2+^ levels at a BK_Ca_ channel coupled with one CaV, steady-state solutions of reaction-diffusion equations solved by CalC approximate the numerical solutions of these equations well, and we confirmed these results in [13], assuming more realistic cases with BK_Ca_ channel coupled with *n* CaVs. In order to get a deeper description of the Ca^2+^ levels, we can exploit compartmental modeling, as performed in our previous work [40], where we characterized the intracellular Ca^2+^ dynamics in glucagon-secreting pancreatic alpha-cells. Compartmental modeling allows us to couple electrical activity and exocytosis, providing a deeper knowledge of the relative contributions of the various sub-cellular compartments (Ca^2+^ nanodomains, sub-membrane compartment, bulk cytosol and endoplasmic reticulum) involved in exocytosis.

## 4. Methods

### 4.1. Electrophysiological Model of Pituitary Cells

The original model of pituitary cells [26] included a single Ca${}^{2+}$ current ($I_{Ca}$), a delayed-rectifier K${}^{+}$ current ($I_{K}$), a Ca${}^{2+}$-gated SK current ($I_{SK}$) and a leak current ($I_{leak}$), in addition to the BK_Ca_ current which was modeled as a purely voltage-dependent current, neglecting Ca^2+^ dependency. We substituted this simplified representation of the BK_Ca_ current with our concise BK_Ca_ model controlled by CaVs in complexes. Then, the dynamics of membrane potential *V* are described by the following ODE:

(6) $$C\frac{dV}{dt}=-\left(I_{Ca}+I_{K}+I_{SK}+I_{BK}+I_{leak}\right),$$

where *C* is the membrane capacitance and $I_{BK}$ is defined by Equation (4) since CaVs do not inactivate. All the other currents are modeled as in [26]. In order to achieve a Hodgkin–Huxley-type model of BK_Ca_ currents (Equation (4)) that take into account local control in BK_Ca_–CaV complexes with different stoichiometries, we first devised a model of a single-channel gating for the BK_Ca_ channel and then we coupled the model with different number of CaVs, each one described by a standard 3-state model, which can be reduced to two states, since CaVs do not inactivate, as reported in the following.

#### 4.1.1. BK_Ca_ Channel Modeling

For describing the BK_Ca_ channel, we used a single-channel gating with two states, as shown in Figure 1A, where *X* corresponds to the closed state and *Y* to the open state. The dynamics of the channel are determined by voltage and calcium-dependent rates, $k^{-}$, and $k^{+}$, describing the transition from the open to close state and from close to open state, respectively. By assuming that, for fixed Ca^2+^ concentration (Ca), BK_Ca_ activity is described by a Boltzmann function [9,27,41] and that the slope parameter of the Boltzmann function is independent of Ca, a reasonable assumption for Ca^2+^ concentrations above 1 μM [10,41,42], as expected in BK_Ca_–CaV complexes [3,10], in [13], we showed that $k^{-}$ and $k^{+}$ can be expressed as a product of voltage and Ca^2+^-dependent terms ($w^{-}\left(V\right)$ and $w^{+}\left(V\right)$ are the voltage dependent rates, $f^{-}\left(Ca\right)$ and $f^{+}\left(Ca\right)$ the Ca^2+^-dependent rates; then, $k^{-}=w^{-}f^{-}$, $k^{+}=w^{+}f^{+}$; see [13] for mathematical expressions). Figure 1B shows the fitting to the experimental data [10], consisting of BK_Ca_ open probabilities and time constants as functions of voltage, at different Ca^2+^ concentrations, by using for $k^{-}$ and $k^{+}$—the optimal parameters estimated in [13].

We also reproduced the dynamics of a more complex model for describing the BK_Ca_ channel proposed by Cox’s lab [10,42]. They assumed that each alpha subunit (four subunits overall), four of which compose the tetrameric structure of the channel [43], has a single Ca^2+^ binding site and a single voltage sensor, and through simplifications, they obtained a 10-state model, where each state can have from zero to four bound Ca^2+^ ions and be open or closed. As shown in [10], the 10-state model is able to reproduce the characteristics and dynamics of the BK_Ca_ channel by fitting BK_Ca_ open probabilities and time constants as functions of voltage, at different Ca^2+^ concentrations. Note that, although the model is complex, it represents an empirical model: individual rate constants are not likely correspond to any real calcium binding events or movements of the channel’s voltage sensors, since real BK_Ca_ contains at least three Ca^2+^ binding sites (a low and two high-affinity calcium binding sites) [43,44,45] and four voltage sensor per subunit [46].

#### 4.1.2. CaV Modeling

We described CaV by using the 3-state ODE model of Figure S1A (see Equations in [13]), where *C* corresponds to the closed state, *O* to the open state and *B* to the inactivated (blocked) state of the calcium channel [47]. α and β represent the voltage-dependent Ca^2+^ channel opening rate and closing rate, respectively, and have the forms as in [47]; γ is a constant reverse reactivation rate and δ represents the Ca^2+^-dependent rate for channel inactivation, which is determined by the Ca^2+^ concentration at the Ca^2+^ sensor, $Ca_{CaV}\left(r\right)$, having the following form by using reaction-diffusion theory [7,10,48]:

(7) $$Ca_{CaV}\left(r\right)=\frac{i_{Ca_{max}}}{8\pi rD_{Ca}F}\exp\left[\frac{-r}{\sqrt{\frac{D_{Ca}}{k_{B}^{+}\left[B_{total}\right]}}}\right]\;.$$

*r* represents the distance of the sensor from the channel pore (in this case r=7 nm) and $i_{Ca_{max}}\;=\;{\bar{g}}_{Ca}\left(V-V_{Ca}\right)$ is the single-channel Ca^2+^ current with ${\bar{g}}_{Ca}$ the single-channel conductance and $V_{Ca}$ the Ca^2+^ reversal potential. The formula defined by Equation (7), called excess buffer approximation (EBA), is based on the assumption that the buffer is unsaturable [48], while another common formula, called rapid buffer approximation (RBA), is valid for buffers that are saturated near an open channel and have Ca^2+^ binding kinetics that are rapid relative to Ca^2+^ diffusion [49,50]. Here, we used EBA, as was done by Cox in his work [10], from which we took the data for our study. Cox found that EBA well approximates the solutions of reaction-diffusion equations solved by the simulation software CalC [51] for computing Ca^2+^ levels at BK_Ca_ channel with the buffer conditions specified in his work, and we confirmed these results in [13] with more realistic cases with BK channel coupled with *n* CaVs. Note that for the single-channel Ca^2+^ current $i_{Ca_{max}}$, we use the same formalism used by Cox (Ohm’s law), although the use of the Goldman–Hodgkin–Katz equation may be more appropriate.

As shown in [47], the processes of activation and inactivation can be approximately separated in time, since activation is much faster than inactivation. In particular, we achieve the following model for the CaV activation variable, $m_{CaV}$,

(8) $$\frac{dm_{CaV}}{dt}=\frac{m_{CaV,\infty }-m_{CaV}}{\tau_{m}}\;,$$

where

(9) $$\begin{array}{cc} m_{CaV,\infty } & =\frac{\alpha }{\alpha +\beta }\;,\;\tau_{m}=\frac{1}{\alpha +\beta }\;, \end{array}$$

and the following equation for inactivation

(10) $$\frac{db}{dt}=m_{CaV,\infty }\delta -\left(m_{CaV,\infty }\delta +\gamma \right)b.$$

Therefore, assuming instantaneous activation, $m_{CaV}=m_{CaV,\infty }$, the 3-state system can be approximated by 1-state ODE model described by Equation (10). Note that we define with *h* the CaV inactivation function, representing the fraction of Ca^2+^ channels not inactivated, h=o+c, where *o* and *c* represent the state variables of 3-state system of Figure S1A. For 1-state model, h=1−b, with *b* given by Equation (10).

We also reproduced a more complex model for describing CaVs, proposed by Cox [10], based on the model of Boland and Bean [52], where the channel is described by a 7-state Markov chain (Figure S1B), with the states $C_{i}$ (i=0,…,4) used for representing the movements of four voltage sensors. Here, the transitions labeled α and β represent the movements of four voltage sensors and are voltage-dependent, having the forms as in [47]. When all four subunits have moved to an activated position, i.e., the channel is in state $C_{4}$, the complex can undergo a final voltage-independent step to the open state *O* with a constant rate ϵ; ζ represents the reverse rate from *O* to $C_{4}$. The inactivation rate δ and the reverse reactivation rate γ have the form as for the 3-state model.

Figure S1C shows the fitting to the data [10] (the red circles in the plots; i.e., the mean values of peak open probabilities (upper plot) and time constants (lower plot)) of the 3-state ODE model (the blue curves) and 7-state Markov chain (the gray curves), both satisfactorily reproducing activation curves and times. For the 3-state ODE model, we used the estimated parameters reported in [13]. Moreover, Figure S1D shows the simulated CaV open probabilities in response to three different voltage steps from −80 mV to −20 (left), 0 (middle) and 20 mV (right), obtained from the 7-state Markov chain model (gray curves), the 3-state ODE model (blue curves) and the corresponding 3-state Markov chain model (dash-dotted black curves), and the 1-state ODE model defined by Equation (10) assuming instantaneous activation $m_{CaV}=m_{CaV,\infty }$ (green curves). The CaV open probabilities for the 3-state ODE model and the corresponding 3-state MC model in response to the different voltage steps approximate the 7-state MC model very well. Note that in response to voltage step from −80 to −20 mV (see left plot in Figure S1D), the 7-state MC model shows an initial delay for the CaV open probability (see dash-dotted gray plot) due to an overestimation of the time constant of the model (compare the gray dash-dotted curve with the red data in the lower plot in Figure 1C for V=−20 mV). Finally, Figure S1F–H shows the whole-cell CaV currents of the different models with different step voltages (Figure S1E). The whole-cell CaV current is defined by using the Hodgkin–Huxley formalism:

(11) $$I_{CaV}=N_{CaV}{\bar{g}}_{Ca}m_{CaV}h_{CaV}\left(V-V_{Ca}\right),$$

where $N_{CaV}=1000$ is the number of CaV channels, each one characterized by a single channel conductance ${\bar{g}}_{Ca}$ (${\bar{g}}_{Ca}=2.8$ pS as in [53] for CaV2.1). $m_{CaV}$ and $h_{CaV}$ are, respectively, the activation and inactivation variables of the channel: for the 3-state ODE model, $m_{CaV}h_{CaV}=o$, where *o* is the state variable corresponding to the open state of the channel; for the simplified 1-state ODE model, assuming instantaneous activation of the CaV currents, $m_{CaV}=m_{CaV,\infty }$ by Equation (9) and $h_{CaV}=1-b$ by solving the single ODE model defined by Equation (10); for the 7-state MC model, $m_{CaV}h_{CaV}=p_{O}$, where $p_{O}$ represents the probability to be in state *O* for the MC of Figure S1B. As for the CaV open probabilities, the 3-state ODE model current approximates the 7-state Markov chain model current very well for each step voltage (Figure S1F,G). Additionally, the further simplification for the 3-state ODE model assuming instantaneous activation of the CaV currents (green plots in Figure S1H) provides a good approximation of the Monte Carlo simulations.

#### 4.1.3. BK_Ca_ Channel Open Probability for BK_Ca_–CaV Complex with 1:1 Stoichiometry

First, we coupled the 2-state model devised for the BK_Ca_ channel with the 3-state model for CaV, resulting in a 6-state MC model for achieving a concise model of whole-cell BK current (Equation (1)) that respects the local effects of BK_Ca_–CaV coupling. Figure 1C shows a cartoon of the model of the devised BK_Ca_–CaV complex with 1:1 stoichiometry: CX, OX and BX correspond to the closed state for the BK_Ca_ channel (*X*) coupled with the closed (*C*), open (*O*) and inactivated (*B*) states for the CaV, respectively, and CY, OY and BY correspond to the open state for the BK_Ca_ channel (*Y*) coupled with the closed (*C*), open (*O*) and inactivated (*B*) states for the CaV, respectively. The parameters $k_{c}^{-}$ and $k_{o}^{-}$ ($k_{c}^{+}$ and $k_{o}^{+}$) are functions of the calcium concentration at the BK_Ca_ channel when the associated CaV is closed, $Ca_{c}$, or inactivated, $Ca_{b}$ (i.e., $Ca_{c}=Ca_{b}$), and to the calcium concentration at the BK_Ca_ channel when the CaV is open, $Ca_{o}$. In particular, the Ca^2+^ levels sensed by the BK_Ca_ channel are assumed to reach steady-state immediately after CaV closure or opening [10]; in this case, $Ca_{c}$ is set equal to 0.2 μM (background Ca^2+^ concentration) and $Ca_{o}$ is given by Equation (7) with r=13 nm representing the distance between CaV and BK_Ca_ channels [3,10]. At V=0 mV, $Ca_{o}\approx 19\;\mu$M. Note that $k_{c}^{+}\approx 0$, since the background Ca^2+^ concentration $Ca_{c}$ is much below the levels needed for BK_Ca_ activation at physiological voltages; i.e., the probability of BK_Ca_ opening when the CaV is closed is practically zero: this approximation is supported by the fact that Ca^2+^ influx via CaVs is needed to open BK_Ca_ channels [54], and that the sub-membrane Ca^2+^ concentration of some hundreds of nM that a BK_Ca_ in a complex without open CaVs would sense, is too low to activate BK_Ca_ channels at physiological voltages [3,10].

Next, we evaluated the dynamics of the deterministic ODE model corresponding to the 6-state MC model. The model is described by the state-variables $p_{Z}$, with Z∈{CX,CY,OX,OY,BX,BY}, representing the probabilities of the complex to be in one of the six states of the model. Then, the BK_Ca_ open probability, $p_{Y}^{\left(1\right)}$, is given as

(12) $$p_{Y}^{\left(1\right)}=p_{CY}+p_{OY}+p_{BY}\;.$$

The 6-state ODE model characterizing $p_{Y}^{\left(1\right)}$ (which can be described by a system of five ODEs because the probabilities sum to 1) can be further simplified by applying timescale analysis. Indeed, the inactivation and reactivation of CaV are slower than (de-)activation; thus, on a fast timescale, the fraction of non-inactivated CaVs ($h=1-(p_{BX}+p_{BY})$) is assumed to be constant, and the model can be split into two submodels with, respectively, four and two states (indicated by green and blue boxes in Figure 1C). Moreover, the 4-state ODE model of the corresponding reduced 4-state MC (green box in Figure 2C) describing the BK_Ca_ channel activation in BK_Ca_–CaV complex with non-inactivated CaV can be further simplified. Indeed, by exploiting quasi-steady state approximation, we obtained a 1-ODE model for describing the BK_Ca_ activation, denoted with $m_{BK}^{\left(1\right)}$ ($m_{BK}^{\left(1\right)}=p_{CY}+p_{OY}$):

(13) $$\frac{dm_{BK}^{\left(1\right)}}{dt}=m_{CaV}k_{o}^{+}-\frac{\left(k_{o}^{+}+k_{o}^{-}\right)\left(k_{c}^{-}+\alpha \right)+\beta k_{c}^{-}}{\alpha +\beta +k_{c}^{-}}m_{BK}=\frac{m_{BK,\infty }^{\left(1\right)}-m_{BK}^{\left(1\right)}}{\tau_{BK}^{\left(1\right)}}\;,$$

with steady-state and time constant given by

(14) $$\tau_{BK}^{\left(1\right)}=\frac{\alpha +\beta +k_{c}^{-}}{\left(k_{o}^{+}+k_{o}^{-}\right)\left(k_{c}^{-}+\alpha \right)+\beta k_{c}^{-}},$$

(15) $$m_{BK,\infty }^{\left(1\right)}=m_{CaV}\;k_{o}^{+}\;\tau_{BK}^{\left(1\right)}.$$

The CaV activation variable $m_{CaV}$ is routinely characterized in patch clamp experiments and included in models of electrical activity via the time-constant, $\tau_{CaV}$, and the steady-state activation function, $m_{CaV,\infty }$ (see Equation (9)). From these quantities, $\alpha =m_{CaV,\infty }/\tau_{CaV}$ and $\beta =1/\tau_{CaV}-\alpha$ can be calculated. Note that Equation (15) makes it explicit how $m_{BK,\infty }$ inherits properties of the associated Ca^2+^ channel type, as has been found experimentally [55,56].

In many whole-cell models (e.g., [26,28,29,30]), the Ca^2+^ currents are assumed to activate instantaneously, which precludes calculation of α and β. Implicitly, such models assume that CaV gating is infinitely faster than the kinetics of other channels in the model. In our setting, this assumption corresponds to investigating the BK_Ca_–CaV model defined by Equations (13)–(15) in the limits α,β→∞. In this case Equations (14) and (15) become

(16) $$\begin{array}{ccc} \tau_{BK}^{\left(1\right)} & \approx & \frac{1}{k_{c}^{-}-m_{CaV,\infty }\left(k_{c}^{-}-k_{o}^{+}-k_{o}^{-}\right)}, \end{array}$$

(17) $$\begin{array}{ccc} m_{BK,\infty }^{\left(1\right)} & \approx & m_{CaV,\infty }\;k_{o}^{+}\;\tau_{BK}^{\left(1\right)}, \end{array}$$

which are completely defined from BK_Ca_ kinetics and $m_{CaV,\infty }$.

By coupling the BK_Ca_ channel activation $m_{BK}^{\left(1\right)}$ defined by Equation (13) with CaV inactivation function *h*, we achieved the BK_Ca_ open probability $p_{Y}^{\left(1\right)}$ of Equation (2) for BK_Ca_–CaV complex with 1:1 stoichiometry.

#### 4.1.4. BK_Ca_ Channel Open Probability for BK_Ca_–CaV Complex with 1:*n* Stoichiometry

BK_Ca_ channels can form ion complexes with more than one CaV with a stoichiometry of 1–4 CaVs channels per BK_Ca_ channel, as experimentally observed [3,4,23]. Here, we introduce a concise but mechanistically correct model of single BK_Ca_–CaV complexes with 1:*n* stoichiometry developed in [13] to be inserted into a whole-cell model of electrical activity.

We extend the 6-state MC model for the complex with 1:1 stoichiometry to incorporate different stoichiometries assuming that *n* CaVs are all located 13 nm from the BK_Ca_ channel [3,10,57]: In this case, the linear buffer approximation is used to compute the Ca^2+^ profile from *n* channels by superimposing *n* nanodomains found for single, isolated CaVs. Then, the MC model of Figure 1C can be extended to a model with 3×n×2 states. However, as discussed in the previous section, we assume that, on a fast timescale, the fraction *h* of non-inactivated CaVs is constant, and note that the BK_Ca_ channel closes rapidly when all CaVs in the complex are inactivated. Hence, for the case of 1:*n* BK_Ca_–CaV stoichiometry, we split the system according to the number *k* of non-inactivated CaVs: the BK_Ca_ activation can be described on fast time scale by the Markov chain model of Figure 2A with 2×(k+1) states, where $C_{k-i}O_{i}X$ and $C_{k-i}O_{i}Y$ correspond to the states with k−i closed and *i* open CaVs, with i=0,…,k, coupled with the closed (*X*) and open (*Y*) BK_Ca_ channels, respectively. As for the case of 1:1 stoichiometry, the dynamics of the BK_Ca_ open probability can be approximated by a single ODE. In particular, we start from the complete ODE system describing the BK_Ca_ channel coupled with *k* non-inactivated CaVs of Figure 2A: the model consists of $2\left(k+1\right)$ ODEs characterizing the state variables $p_{C_{k-i}O_{i}X}$ and $p_{C_{k-i}O_{i}Y}$, corresponding to the probability of having k−i closed and *i* open CaVs, with i=0,…,k, coupled with the closed (*X*) and open (*Y*) BK_Ca_ channels, respectively. The activation of the BK_Ca_ surrounded by *k* non-inactivated CaVs, denoted with $m_{BK}^{\left(k\right)}$, is then

(18) $$m_{BK}^{\left(k\right)}=p_{C_{k}Y}+\sum_{i=1}^{k-1}p_{C_{k-i}O_{i}Y}+p_{O_{k}Y}\;,$$

By taking into account that

(19) $$\begin{array}{ccc} p_{C_{k}X} & = & {\left(1-m_{CaV}\right)}^{k}-p_{C_{k}Y} \end{array}$$

(20) pCk−iOiX=ki1−mCaVk−imCaVi−pCk−iOiY,i=1,…,k−1

(21) $$\begin{array}{ccc} p_{O_{k}X} & = & m_{CaV}^{k}-p_{O_{k}Y} \end{array}$$

and renaming the state variables as follows

(22) $$\begin{array}{ccc} p_{Y_{0}} & = & p_{C_{k}Y} \end{array}$$

(23) $$\begin{array}{ccc} p_{Y_{1}} & = & p_{C_{k-1}OY}+p_{Y_{0}} \end{array}$$

⋮

(24) $$\begin{array}{ccc} p_{Y_{i}} & = & p_{C_{k-i}O_{i}Y}+p_{Y_{i-1}} \end{array}$$

⋮

(25) $$\begin{array}{ccc} m_{BK}^{\left(k\right)}=p_{Y_{k}} & = & p_{O_{k}Y}+p_{Y_{k-1}}, \end{array}$$

the ODE system is reduced from 2(k+1) to (k+1) equations.

Moreover, assuming the quasi-steady state approximation for $p_{Y_{i}}$, with i=0,…,k−1, the ODE system of (k+1) equations is reduced to the following single ODE, describing the dynamics of the BK_Ca_ activation with *k* non-inactivated CaVs:

(26) $$\frac{dm_{BK}^{\left(k\right)}}{dt}=\frac{m_{BK,\infty }^{\left(k\right)}-m_{BK}^{\left(k\right)}}{\tau_{BK}^{\left(k\right)}},$$

where $m_{BK,\infty }^{\left(k\right)}$ and $\tau_{BK}^{\left(k\right)}$ are explicit functions of *V*, directly or via the local Ca^2+^ concentration (see Equation (S36) in the Supporting Material of [13]).

By assuming instantaneous activation of CaVs, the model defined by Equation (26) can be further simplified. Indeed, in this case (i.e., $m_{CaV}=m_{CaV,\infty }$) the vertical transitions in Figure 2A are in quasi-equilibrium, and then

(27) pCk−iOiY=ki(1−mCaV,∞)k−imCaV,∞ipY,i=0,…,k,

and $m_{BK}^{\left(k\right)}$ follows Equation (26) with

(28) τBK(k)=∑i=1kki(1−mCaV,∞)k−imCaV,∞i(koi++koi−)+(1−mCaV,∞)kkc−−1,

(29) mBK,∞(k)=∑i=1kki(1−mCaV,∞)k−imCaV,∞ikoi+τBK(k).

By taking into account that the probability that *k* non-inactivated CaVs are present in a complex with *n* CaVs is nkhk(1−h)n−k, we obtain Equation (3) to describe the BK_Ca_ channel open probability for BK_Ca_–CaV complex with 1:*n* stoichiometry.

### 4.2. Exocytosis Model

We modeled the single granule, adjacent to the plasma membrane and primed for exocytosis, as performed in [22], by the Markov chain shown in Figure 3A. The granule can be in one of four different states depending on the number of Ca^2+^ ions bound to the Ca^2+^ sensor on the granule, likely synaptotagmin [58]: in $G_{0}$ with no bound Ca^2+^ ions, in $G_{1}$ with one, in $G_{2}$ with two or in $G_{3}$ with three bound ions. Once it is in $G_{3}$, the granule can fuse with the membrane and release its hormone content, assuming the final state *Y* [25,59]. Therefore, the model takes values in the state space $S=\{G_{0},G_{1},G_{2},G_{3},Y\}$ and its transition rate or generator matrix $M_{G}$ is given by

(30) $$\begin{array}{c} M_{G}=\left[\begin{array}{ccccc} -3\;k_{Ca} & 3\;k_{Ca}\; & 0 & 0 & 0\\ k_{-} & -2\;k_{Ca}-k_{-} & 2\;k_{Ca} & 0 & 0\\ 0 & 2\;k_{-} & -k_{Ca}-2\;k_{-} & k_{Ca} & 0\\ 0 & 0 & 3\;k_{-} & -u-3\;k_{-} & u\\ 0 & 0 & 0 & 0 & 0 \end{array}\right]\;, \end{array}$$

where

(31) $$k_{Ca}=k_{+}\times \left[Ca_{CaV}\left(r_{G}\right)\right]$$

represents the Ca^2+^ binding rate, with $Ca_{CaV}\left(r_{G}\right)$, the Ca^2+^ concentration at the granule sensor, given by Equation (7) with $r=r_{G}$ being the distance from the CaV to the Ca^2+^ sensor on the granule. In this paper, the distance from the CaV to the granule means the distance from the CaV to the Ca^2+^ sensor on the granule, which will be of the order of tens of nm. For comparison, secretory granules have diameters on the order 100–500 nm [60,61,62,63]. We assumed a constant number of Ca^2+^ sensor molecules, which was, therefore, included in the binding parameter $k_{Ca}$. The parameter $k_{-}$ was the unbinding rate, and *u* was the fusion rate. The values for $k_{-}$, $k_{+}$ and *u* were equal to those used in [22]. Note that, since $G_{3}$ dynamics are fastest (the value of *u* is much higher than those of the other parameters), the 5-state MC model can be reduced to a 4-state MC model, where the dynamics of states $G_{2}$ and $G_{3}$ can be described by an auxiliary variable $G_{23}$, using quasi-steady state approximation for the corresponding ODE model, as performed in [22]. Here, we exploit the complete sequence of the 5-state MC model in order to reproduce the delayed exocytosis with respect to a raise in the Ca^2+^ concentration, as observed by flash-release experiments [36,37,38], although the 4-state MC model does not modify the results substantially. Instead, a further state-reduction can significantly modify the exocytosis probability.

By coupling the 5-state exocytosis model with the 3-state model of Figure S1A for CaV, we obtained the 15-state MC model of Figure 3B. For the general case, where the granule is coupled with *n* CaVs, we obtain a $\left(5\times n\times 3\right)$-state MC model (see [22] for model details and mathematical description). For the electrical activity model in pituitary cells described by Equation (6), CaVs do not inactivate, and then, each CaV can be described by single-channel gating with two states, closed, *C*, and open, *O*, and the granule release by $5\left(n+1\right)$-state MC model, as shown in Figure 3C.

## Supplementary Materials

The following are available online at https://www.mdpi.com/1422-0067/20/23/6057/s1, Figure S1: CaV modeling.

## References

[1] Hodgkin A.L., Huxley A.F. (1952). A quantitative description of membrane current and its application to conduction and excitation in nerve. J. Physiol..

[2] Anderson D., Mehaffey W.H., Iftinca M., Rehak R., Engbers J.D.T., Hameed S., Zamponi G.W., Turner R.W. (2010). Regulation of neuronal activity by Cav3-Kv4 channel signaling complexes. Nat. Neurosci..

[3] Berkefeld H., Fakler B., Schulte U. (2010). Ca^2+^-Activated K^+^ Channels: From Protein Complexes to Function. Physiol. Rev..

[4] Suzuki Y., Yamamura H., Ohya S., Imaizumi Y. (2013). Caveolin-1 Facilitates the Direct Coupling between Large Conductance Ca^2+^-activated K^+^(BKCa) and Cav1.2 Ca^2+^ Channels and Their Clustering to Regulate Membrane Excitability in Vascular Myocytes. J. Biol. Chem..

[5] Chad J., Eckert R. (1984). Calcium domains associated with individual channels can account for anomalous voltage relations of CA-dependent responses. Biophys. J..

[6] Simon S., Llinás R. (1985). Compartmentalization of the submembrane calcium activity during calcium influx and its significance in transmitter release. Biophys. J..

[7] Neher E. (1998). Vesicle Pools and Ca^2+^ Microdomains: New Tools for Understanding Their Roles in Neurotransmitter Release. Neuron.

[8] Fakler B., Adelman J.P. (2008). Control of KCa Channels by Calcium Nano/Microdomains. Neuron.

[9] Berkefeld H., Fakler B. (2013). Ligand-Gating by Ca^2+^ Is Rate Limiting for Physiological Operation of BKCa Channels. J. Neurosci..

[10] Cox D.H. (2014). Modeling a Ca^2+^ Channel/BK_Ca_ Channel Complex at the Single-Complex Level. Biophys. J..

[11] Buchholz P., Kriege J., Felko I. (2014). Phase-Type Distributions. Input Modeling with Phase-Type Distributions and Markov Models.

[12] Segel L.A., Slemrod M. (1989). The Quasi-Steady-State Assumption: A Case Study in Perturbation. SIAM Rev..

[13] Montefusco F., Tagliavini A., Ferrante M., Pedersen M.G. (2017). Concise Whole-Cell Modeling of BK_Ca_-CaV Activity Controlled by Local Coupling and Stoichiometry. Biophys. J..

[14] Williams G.S., Huertas M.A., Sobie E.A., Jafri M.S., Smith G.D. (2007). A Probability Density Approach to Modeling Local Control of Calcium-Induced Calcium Release in Cardiac Myocytes. Biophys. J..

[15] Burgoyne R.D., Morgan A. (2003). Secretory Granule Exocytosis. Physiol. Rev..

[16] Barg S. (2003). Mechanisms of Exocytosis in Insulin-Secreting Beta-Cells and Glucagon-Secreting Alpha-Cells. Pharmacol. Toxicol..

[17] Jahn R., Hanson P.I. (1998). SNAREs line up in new environment. Nature.

[18] Thorn P., Zorec R., Rettig J., Keating D.J. (2016). Exocytosis in non-neuronal cells. J. Neurochem..

[19] Pedersen M.G., Tagliavini A., Cortese G., Riz M., Montefusco F. (2017). Recent advances in mathematical modeling and statistical analysis of exocytosis in endocrine cells. Math. Biosci..

[20] Bornschein G., Schmidt H. (2019). Synaptotagmin Ca^2+^ Sensors and Their Spatial Coupling to Presynaptic Cav Channels in Central Cortical Synapses. Front. Mol. Neurosci..

[21] Gandasi N.R., Yin P., Riz M., Chibalina M.V., Cortese G., Lund P.E., Matveev V., Rorsman P., Sherman A., Pedersen M.G. (2017). Ca^2+^ channel clustering with insulin-containing granules is disturbed in type 2 diabetes. J. Clin. Investig..

[22] Montefusco F., Pedersen M.G. (2018). Explicit Theoretical Analysis of How the Rate of Exocytosis Depends on Local Control by Ca^2+^ Channels. Comput. Math. Methods Med..

[23] Vivas O., Moreno C.M., Santana L.F., Hille B. (2017). Proximal clustering between BK and CaV1.3 channels promotes functional coupling and BK channel activation at low voltage. eLife.

[24] Stojilkovic S.S. (2005). Ca^2+^-regulated exocytosis and SNARE function. Trends Endocrinol. Metab. TEM.

[25] Tagliavini A., Tabak J., Bertram R., Pedersen M.G. (2016). Is bursting more effective than spiking in evoking pituitary hormone secretion? A spatiotemporal simulation study of calcium and granule dynamics. Am. J. Physiol.-Endocrinol. Metab..

[26] Tabak J., Toporikova N., Freeman M.E., Bertram R. (2006). Low dose of dopamine may stimulate prolactin secretion by increasing fast potassium currents. J. Comput. Neurosci..

[27] Berkefeld H., Sailer C.A., Bildl W., Rohde V., Thumfart J.O., Eble S., Klugbauer N., Reisinger E., Bischofberger J., Oliver D. (2006). BKCa-Cav Channel Complexes Mediate Rapid and Localized Ca^2^-Activated K^+^ Signaling. Science.

[28] Roper P., Callaway J., Shevchenko T., Teruyama R., Armstrong W. (2003). AHP’s, HAP’s and DAP’s: How Potassium Currents Regulate the Excitability of Rat Supraoptic Neurones. J. Comput. Neurosci..

[29] Pedersen M.G. (2010). A Biophysical Model of Electrical Activity in Human Beta-Cells. Biophys. J..

[30] Riz M., Braun M., Pedersen M.G. (2014). Mathematical Modeling of Heterogeneous Electrophysiological Responses in Human Beta-Cells. PLoS Comput. Biol..

[31] Pallotta B.S., Magleby K.L., Barrett J.N. (1981). Single channel recordings of Ca^2+^-activated K^+^ currents in rat muscle cell culture. Nature.

[32] Benton M.D., Lewis A.H., Bant J.S., Raman I.M. (2013). Iberiotoxin-sensitive and -insensitive BK currents in Purkinje neuron somata. J. Neurophysiol..

[33] Storm J.F. (1987). Action potential repolarization and a fast after-hyperpolarization in rat hippocampal pyramidal cells. J. Physiol..

[34] Braun M., Ramracheya R., Bengtsson M., Zhang Q., Karanauskaite J., Partridge C., Johnson P.R., Rorsman P. (2008). Voltage-Gated Ion Channels in Human Pancreatic Beta-Cells: Electrophysiological Characterization and Role in Insulin Secretion. Diabetes.

[35] Houamed K.M., Sweet I.R., Satin L.S. (2010). BK channels mediate a novel ionic mechanism that regulates glucose-dependent electrical activity and insulin secretion in mouse pancreatic beta-cells. J. Physiol..

[36] Barg S., Ma X., Eliasson L., Galvanovskis J., Göpel S.O., Obermüller S., Platzer J., Renström E., Trus M., Atlas D. (2001). Fast exocytosis with few Ca^2+^ channels in insulin-secreting mouse pancreatic beta-cells. Biophys. J..

[37] Barg S., Galvanovskis J., Gopel S.O., Rorsman P., Eliasson L. (2000). Tight coupling between electrical activity and exocytosis in mouse glucagon-secreting alpha-cells. Diabetes.

[38] Voets T., Neher E., Moser T. (1999). Mechanisms Underlying Phasic and Sustained Secretion in Chromaffin Cells from Mouse Adrenal Slices. Neuron.

[39] Milescu L.S., Yamanishi T., Ptak K., Mogri M.Z., Smith J.C. (2008). Real-Time Kinetic Modeling of Voltage-Gated Ion Channels Using Dynamic Clamp. Biophys. J..

[40] Montefusco F., Pedersen M.G. (2015). Mathematical modelling of local calcium and regulated exocytosis during inhibition and stimulation of glucagon secretion from pancreatic alpha-cells. J. Physiol..

[41] Latorre R., Brauchi S. (2006). Large conductance Ca2+-activated K+ (BK) channel: Activation by Ca2+ and voltage. Biol. Res..

[42] Cox D., Cui J., Aldrich R. (1997). Allosteric Gating of a Large Conductance Ca-activated K^+^ Channel. J. Gen. Physiol..

[43] Bao L., Rapin A.M., Holmstrand E.C., Cox D.H. (2002). Elimination of the BKCa Channel’s High-Affinity Ca^2+^ Sensitivity. J. Gen. Physiol..

[44] Xia X.M., Zeng X., Lingle C.J. (2002). Multiple regulatory sites in large-conductance calcium-activated potassium channels. Nature.

[45] Yusifov T., Savalli N., Gandhi C.S., Ottolia M., Olcese R. (2007). The RCK2 domain of the human BKCa channel is a calcium sensor. Proc. Natl. Acad. Sci. USA.

[46] Horrigan F.T., Aldrich R.W. (1999). Allosteric Voltage Gating of Potassium Channels II. J. Gen. Physiol..

[47] Sherman A., Keizer J., Rinzel J. (1990). Domain model for Ca^2+^-inactivation of Ca^2+^ channels at low channel density. Biophys. J..

[48] Neher E. (1986). Concentration profiles of intracellular Ca^2+^ in the presence of diffusible chelator. Calcium Electrogenesis and Neuronal Functioning, Exp. Brain Res. 14.

[49] Naraghi M., Neher E. (1997). Linearized buffered Ca^2+^ diffusion in microdomains and its implications for calculation of [Ca^2+^] at the mouth of a calcium channel. J. Neurosci. Off. J. Soc. Neurosci..

[50] Sherman A., Smith G.D., Dai L., Miura R.M. (2001). Asymptotic Analysis of Buffered Calcium Diffusion near a Point Source. SIAM J. Appl. Math..

[51] Matveev V., Zucker R.S., Sherman A. (2004). Facilitation through Buffer Saturation: Constraints on Endogenous Buffering Properties. Biophys. J..

[52] Boland L.M., Bean B.P. (1993). Modulation of N-type calcium channels in bullfrog sympathetic neurons by luteinizing hormone-releasing hormone: Kinetics and voltage dependence. J. Neurosci. Off. J. Soc. Neurosci..

[53] Weber A.M., Wong F.K., Tufford A.R., Schlichter L.C., Matveev V., Stanley E.F. (2010). N-type Ca^2+^ channels carry the largest current: implications for nanodomains and transmitter release. Nat. Neurosci..

[54] Marcantoni A., Vandael D.H.F., Mahapatra S., Carabelli V., Sinnegger-Brauns M.J., Striessnig J., Carbone E. (2010). Loss of Cav1.3 Channels Reveals the Critical Role of L-Type and BK Channel Coupling in Pacemaking Mouse Adrenal Chromaffin Cells. J. Neurosci..

[55] Berkefeld H., Fakler B. (2008). Repolarizing Responses of BKCa-Cav Complexes Are Distinctly Shaped by Their Cav Subunits. J. Neurosci..

[56] Rehak R., Bartoletti T.M., Engbers J.D.T., Berecki G., Turner R.W., Zamponi G.W. (2013). Low Voltage Activation of KCa1.1 Current by Cav3-KCa1.1 Complexes. PLoS ONE.

[57] Muller A., Kukley M., Uebachs M., Beck H., Dietrich D. (2007). Nanodomains of Single Ca^2+^ Channels Contribute to Action Potential Repolarization in Cortical Neurons. J. Neurosci..

[58] Pinheiro P.S., Houy S., Sørensen J.B. (2016). C2-domain containing calcium sensors in neuroendocrine secretion. J. Neurochem..

[59] Voets T. (2000). Dissection of Three Ca^2+^-Dependent Steps Leading to Secretion in Chromaffin Cells from Mouse Adrenal Slices. Neuron.

[60] Grabner C.P., Price S.D., Lysakowski A., Fox A.P. (2005). Mouse Chromaffin Cells Have Two Populations of Dense Core Vesicles. J. Neurophysiol..

[61] Andersson S.A., Pedersen M.G., Vikman J., Eliasson L. (2011). Glucose-dependent docking and SNARE protein-mediated exocytosis in mouse pancreatic alpha-cell. Pflügers Arch. Eur. J. Physiol..

[62] Olofsson C.S., Göpel S.O., Barg S., Galvanovskis J., Ma X., Salehi A., Rorsman P., Eliasson L. (2002). Fast insulin secretion reflects exocytosis of docked granules in mouse pancreatic beta-cells. Pflügers Arch..

[63] Dean P.M. (1973). Ultrastructural morphometry of the pancreatic beta-cell. Diabetologia.

