# Supplementary material for: From Local to Global Modeling for Characterizing Calcium Dynamics and Their Effects on Electrical Activity and Exocytosis in Excitable Cells
Source: Int J Mol Sci. 2019 Nov 30;20(23):6057. doi: 10.3390/ijms20236057 (PMC6928823; doi:10.3390/ijms20236057)
Supplement: Supplementary file 1 [file ijms-20-06057-s001.pdf]

Supplementary Material

From local to global modeling for characterizing calcium dynamics and their effects on electrical activity and exocytosis in excitable cells

Francesco Montefusco, Morten G. Pedersen

List of contents

- Supplementary Figure
  - Figure S1 . . . . . . p. 2

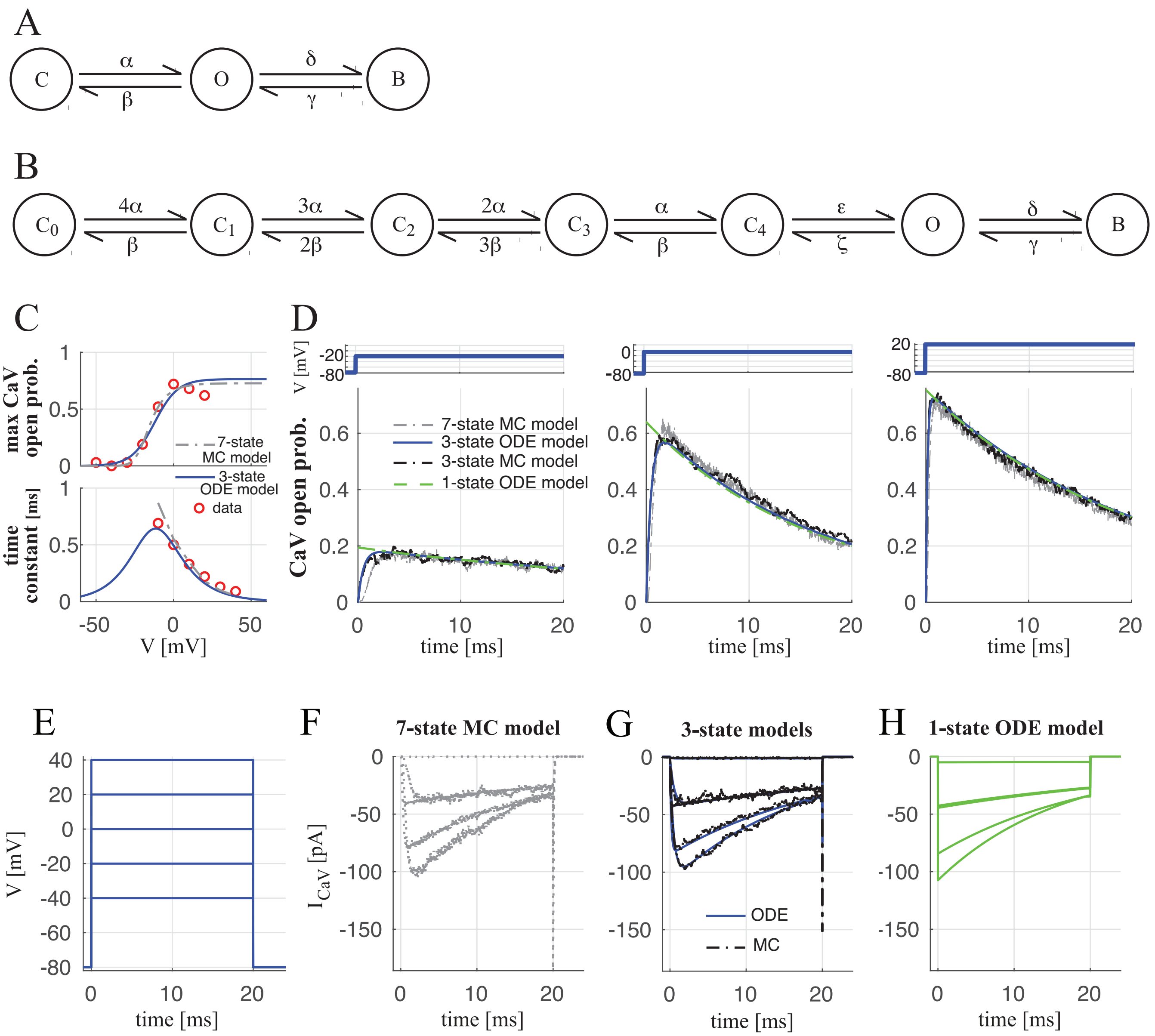

Figure S1: CaV modeling: (**A**) A 3-state model for a CaV where C is the closed state, O the open state and B the inactivated or blocked state. (**B**) A 7-state model for a CaV where C_i are the closed states representing the four voltage subunits: C_0 with no active subunits, C_1 with one and so on, C_4 with all the four subunits in active state and ready to reach the open state O. The CaV inactivation is described as for panel **A**. (**C**) Experimental data (red circles) presented by Cox [10] from HEK293 cell expressing CaV2.1: the upper and lower plots reports the mean values of peak open probabilities and time constants as function of voltage, respectively. The blue solid curves represent the best fit to the data obtained by 3-state ODE model reported in panel **A**. The grey dash-dotted curves represent the fit to the data obtained by 7-state MC model proposed by Cox [10] and described in panel **B**. (**D**) Simulated CaV open probabilities in response to three different voltage steps from -80 mV to -20 (left plot), 0 (middle) and 20 mV (right), obtained from the 7-state Markov chain model (gray curves), the 3-state ODE model (blue curves) and the corresponding 3-state Markov chain model (dash-dotted black curves), and the 1-state ODE model assuming instantaneous CaV activation (green curves). (**E**) Voltage steps from -80 mV to from -40 mV to 40 mV in 20 mV increments for 20 ms and then back to -80 mV. Simulated CaV currents in response to the different voltage steps, shown in panel **E**, obtained from the 7-state Markov chain model **(F)**, the 3-state ODE (blue curves) and MC (black curves) models (**G**), and the corresponding 1-state ODE model assuming instantaneous CaV activation (**H**). In (**D**), (**F**) and (**G**) one-thousand Monte Carlo simulations were performed for the Markov chain models and the average of these Monte Carlo simulations is shown.
